# Patient Education on Oral Anticoagulation

**DOI:** 10.3390/pharmacy6020034

**Published:** 2018-04-20

**Authors:** Emily M Hawes

**Affiliations:** 1 University of North Carolina Eshelman School of Pharmacy, Chapel Hill, NC 27514, USA; emily_hawes@med.unc.edu; 2 University of North Carolina School of Medicine, Chapel Hill, NC 27599, USA

**Keywords:** pharmacy, anticoagulation, patient education, counseling, communication

## Abstract

Given the potential harm associated with anticoagulant use, patient education is often provided as a standard of care and emphasized across healthcare settings. Effective anticoagulation education involves face-to-face interaction with a trained professional who ensures that the patient understands the risks involved, the precautions that should be taken, and the need for regular monitoring. The teaching should be tailored to each patient, accompanied with written resources and utilize the teach-back method. It can be incorporated in a variety of pharmacy practice settings, including in ambulatory care clinics, hospitals, and community pharmacies.

## 1. Introduction

Anticoagulants, which are considered “high alert medications,” can often lead to adverse drug events in the inpatient and outpatient healthcare setting if not managed appropriately. High alert medications refer to drugs that have an increased risk of causing significant harm when used in error [1]. Many of anticoagulation-associated adverse effects result from medication errors, suggesting they are preventable [2]. Therefore, national patient safety goals for the Joint Commission emphasize decreasing the possibility of patient harm due to anticoagulants (including apixaban, dabigatran, edoxaban, rivaxoraban, and warfarin) and recommend accurate and accessible patient education [3].

## 2. Anticoagulation Management Services

The majority of both inpatient and outpatient anticoagulation management services (AMS) utilize providers with anticoagulation expertise and incorporate patient and outcome tracking, comprehensive patient education, and quality improvement (QI) activities [4]. Studies have consistently demonstrated improved clinical outcomes with AMS compared to usual care [5]. With disease-state and medication knowledge regarding anticoagulation, pharmacists are well-suited to manage AMS. Studies have reported that patients who receive pharmacist-led warfarin management services whether in the community, inpatient or outpatient setting achieve significantly better International Normalized Ratio (INR) control compared with those patients who receive usual care [6,7,8,9,10,11,12,13,14]. In addition to achieving therapeutic INR range, pharmacist-led anticoagulation management has also been found to reduce adverse events associated with anticoagulation, resulting in both decreased hospitalizations and decreased hospital length of stay [15]. Furthermore, these services have been shown to reduce the rates of anticoagulation-related emergency department (ED) visits and hospitalizations, with significant financial impact. In a 2010 study, pharmacist-managed services averted $141,277 in hospitalization costs and $10,183 in ED costs versus a nurse-managed service, and $95,579 in hospitalization costs and $5511 in ED costs compared with usual care [16].

Even less comprehensive methods, such as a single patient counseling session at discharge, have noted positive outcomes. Enhanced patient understanding of warfarin has resulted in better INR control and decreased hospital readmission rates [17,18,19]. In one study, patients who did not receive pharmacist education in the hospital prior to discharge required more interventions related to adherence concerns, incorrect administration, and continued use of interacting drugs versus those who did (36.4% vs. 12.9%, *p* = 0.0005). In the same population, patients who had not received pharmacist counseling had higher readmission rates and ED visits due to anticoagulation problems within 3 months of discharge (12.12% vs. 1.85%, *p* = 0.0069) [19]. In the inpatient setting, patient education resulted in significantly reduced interacting medications, extreme INRs, and adverse events during warfarin therapy [20]. Wang et al. highlighted patients’ concerns and deficits in knowledge regarding warfarin treatment, and also demonstrated their association with warfarin adherence and INR control. Patients had inadequate understanding of warfarin-diet and warfarin-drug interactions. The most common concerns regarding taking warfarin were related to warfarin-drug interactions (36.1%), forgetting to take warfarin (26.2%) and concerns about adverse effects (25.7%) [21]. Pharmacist-managed warfarin services, which includes patient education, have shown positive outcomes with respect to safety, efficacy, and cost savings.

Thus, some institutions have implemented AMS for both patients receiving warfarin as well as the direct oral anticoagulants (DOAC). For DOAC management, it is not clearly defined when and how to best provide patient education, how often to evaluate for bleeding or thrombosis, or how often to screen for interacting medications and changes in organ function [22,23,24,25,26]. In patients on DOACs, it is recommended to regularly assess for changes in organ function and evaluate for clinically-relevant drug interactions [27,28,29,30,31,32]. A recent study found that older age and higher number of concomitant medications were associated with higher DOAC adherence. Predictors of lower adherence were higher number of comorbidities and being a naïve anticoagulant (AC) user (no prior AC use). Prior exposure and management in anticoagulation clinics increases patient understanding of anticoagulation and the potential consequences of noncompliance. Therefore, these factors may lead to increased patient motivation to adhere to DOAC therapy [22].

According to the Joint Commission, education should be provided regarding anticoagulant therapy to prescribers, staff, patients, and families. Effective anticoagulation patient education involves face-to-face interaction with a trained professional who ensures that the patient understands the risks involved, the precautions that should be taken, and the need for regular monitoring [5]. Anticoagulation education is often provided as a standard of care and emphasized across healthcare settings [5,30]. Although the majority of research is associated with counseling in the ambulatory care clinic or hospital setting, patient education can be implemented in any setting, including at the community pharmacy [13,14].

## 3. Patient Counseling

Effective medication counseling can empower patients to be active partners in their care and enhance treatment compliance. Studies demonstrate that patients who are engaged in their health have enhanced care experiences, better outcomes and reduced overall healthcare costs [33,34]. Establishing a therapeutic relationship built on trust can be critical to promoting understanding and empowering self-management. This mutually beneficial exchange in which the patient gives authority to the provider and the provider gives information and commitment to the patient is central to effective medication management [35,36,37]. Patients should be empowered as partners in their care, with appropriate teaching and resources. Education involves assessing the patient’s understanding about his or her health problems and medications, the ability to use the prescribed medications correctly, and attitudes toward the health-related issues and associated pharmacotherapy [24,35,36].

Asking open-ended questions is a method that can be used to evaluate patient understanding, reinforce key concepts, and decide what information is needed for patients. For example, “what questions do you have for me?” versus “do you have any questions?” can invite richer conversation [36]. When initiating a new drug, an inquiry about each medication’s indications, the patient’s expectations and asking the patient to show self-administration can promote understanding. This methodology can be repeated during follow up visits, to identify medication-related problems or concerns that arise.

Visual aids and demonstration devices can promote patient understanding. Opening pill bottles, for instance, can emphasize the medication color, size, and shape to the patient. For injectable medications, this may comprise showing patients the exact marking on the measuring devices to ensure accurate dosing. Devices such as low-molecular weight heparin syringes may necessitate a demo of the assembly and correct administration. The direct observation of drug-use can also reveal accurate usage and strengthen teaching of important points. Patient-friendly written resources as an adjunct to verbal communication can also help improve patient awareness [35,36]. In fact, multiple modalities of education, such as verbal, written and video should be used to emphasize important points. The combination of education methods improves patient and/or caregiver knowledge and satisfaction, but this is not always done [38]. According to an ISMP survey, 25% of nurses note that they do not provide written materials to accompany verbal education to patients about their medications [39]. Unfortunately, drug information sources are often inconsistent, complex, incomplete, unavailable, and written at a college reading level or not available in the patient’s language [35,39]. Creation of a medication list, using graphics or simple phrases to show the medicine, its indication, how much to take, and when to take it can be useful resource [40,41].

Understanding patients’ cultural background, especially health and illness beliefs, attitudes, and practices can help tailor educational strategies. Health care professionals should adjust their content and style to patients’ communication skills, often with the use of teaching aids, interpreters, or cultural guides. Assessing a patient’s cognitive abilities, health literacy, learning style, and physical status can also help individualize the educational method to meet the patient’s needs. Some patients may learn best by listening to information, by seeing a picture or model, and/or by touching the pills and devices [36,40]. 

Some patients may lack the visual ability to correctly read prescription labels on bottles, find syringe markings, or follow written instructions. An impaired ability to read information on medication bottles or package inserts increases the likelihood for self-management errors. These patients may benefit from services such as blister packaging by community pharmacies. In addition, they may rely on family members or caregivers to read instructions, memorize how the pill feels in their hand, or use enhanced lighting devices and magnifiers. Other patients may use technology (such as talking pill bottles or home INR devices) or computer software that converts printed information to Braille. Promoting the use of a weekly pill box and encouraging patients to bring it to clinic appointments can help improve adherence and can assist the provider in confirming that the patient is organizing medications as prescribed [35,36]. 

Functional limitations can reduce patient dexterity or strength which makes it challenging to open child-resistant containers, and may require special lids for bottles. Patients may also have hearing difficulties which reduces understanding of oral education and forces reliance on a written instructions. Challenges in verbal communication between providers and patients can also lead to mistakes in the execution of the prescribed regimen. Although approaches for meeting the medication needs of patients with hearing or visual impairment are challenging, efforts should be made to tailor self-management to each patient’s limitations [34].

Medication self-management requires physical and cognitive skills, including higher-level cortical processing and integration. With cognitive impairment, parts of the brain responsible for thinking and executive functions (such as memory, reasoning, learning) can be diminished and may interfere with self-management of medications. Even memory changes associated with normal aging can impair effective drug use. Behavior modification, caregiver education and support, and utilizing adherence tools such as weekly pill boxes, can assist in improved management of medications in patients with cognitive impairment [35,40]. The education level and patients’ knowledge can impact the global management of the anticoagulation [42]. Thus, every effort should be made to clearly educate and evaluate understanding. When interacting with patients, health care providers should explain concepts clearly without using medical jargon. Terms such as use vs. utilize, side effect vs. adverse reaction, when you need it vs. PRN, and by mouth vs. oral are often easier to understand for patients [40]. Standardized terminology about dosing schedules (e.g., morning, noon, night, and bedtime) improves understanding and reduces administration errors. Imprecise information about dosing frequency (e.g., every 4 to 6 h) should be avoided for those patients with low health literacy. A prescription label that has explicit instructions such as “Take one tablet in the morning and one at 5 PM” instead of “Take one tablet twice daily” decreases the possibility of improper dosing frequency and administration. For a patient taking rivaroxaban for atrial fibrillation, including instructions to “Take rivaroxaban once a day with your evening meal” is more specific than “Take rivaroxaban once a day with food” [40,41]. Providers should be mindful of the pace and content and volume of speech, especially when communicating to patients with limited health literacy. Key information should be repeated with succinct explanations [40,41].

A “teach back” technique is an effective way to evaluate patient understanding, clarify key points, and remove any communication gaps between the patient and health educator. In this approach, patients are asked to repeat instructions in their own words to confirm understanding. A health care professional, for example, may say something as follows: “I want to make sure that I have explained everything clearly. If you were trying to explain to your partner how to take this medication, what would you say [24,40]? If a patient cannot accurately repeat what was presented, the information is clarified, and the patient is invited to teach back again. This process continues until the patient can accurately describe the directions [36,40]. The teach back may be an effective strategy to identify errors in drug administration, since studies have found a gap between a patient’s ability to verbalize instructions correctly, and his or her ability to accurately show the correct number of pills to be taken daily [40,41].

## 4. Anticoagulation Information

The medication counseling session should ideally include the information listed below, and can be modified based on each patient’s anticoagulant and monitoring plan and the educator’s clinical judgement [23,24,36,37,43].

- The drug’s brand and generic name and, when needed, its therapeutic class
- The drug’s purpose and how it pertains to thrombus formation
- The drug’s anticipated onset and what to do if the expected result does not occur
- The drug’s route, dosage form, dose, frequency, and duration of treatment
- Directions for preparing and using the drug (such as low-molecular weight heparin)
- Missed-dose management
- Precautions to be aware of when using the drug and the potential measures to decrease bleeding risk and trauma
- Common side effects that may occur (including signs and symptoms of bleeding) and steps to follow if they occur, actions to prevent or reduce their occurrence, and what to do if they occur, including when to notify a healthcare professional
- Strategies for self-monitoring and the importance of regular monitoring to reduce bleeding and thrombosis
- Potential drug–drug (including OTC), drug–food, and drug–disease interactions or contraindications
- Need to inform provider if you are pregnant or plan to become pregnant
- Need to inform provider before a procedure or hospitalization
- Need to notify all health care providers of use
- Need to wear medical identification
- Importance of not stopping without consulting health care provider
- Need to consult health care provider before starting any new drug
- Inform provider of all medication changes, including over-the-counter and herbals
- Importance of taking exactly as prescribed and use of an adherence aid if needed
- Prescription refills authorized and the process for obtaining refills
- Proper drug storage and disposal
- Other helpful information unique to the specific patient or therapy

According the Joint Commission, anticoagulation education should include the necessity of follow-up monitoring, adherence, drug-food interactions, and the potential for side effects and drug interactions [5]. Effort should be made to integrate patient-centered educational methods to promote understanding of how to handle high-risk situations that may compromise safety related to anticoagulation [43]. There are some similarities in counseling a patient on DOAC and warfarin, but some important differences need to be noted. 

## 5. Specific Drug Considerations

Anticoagulant counseling should be tailored to each patient, medication specific, and at an appropriate literacy level. Table 1 includes practical considerations for each of the oral anticoagulants. As the number of indications and data supporting DOAC use expands, educational tools need to be relied on and updated frequently. An important patient education point is that DOACs should not be discontinued unless specifically directed by a healthcare professional because of the rapid decline of protective anticoagulation that can occur (within 12–24 h after the last dose). The necessity for strict adherence should be implicitly explained to patients on DOACs. DOACs have a very predictable anticoagulant effect and monitoring of coagulation assays is not routinely required to guide therapy. This could wrongly lead some patients to determine that no follow up is needed. Patients should be educated regarding the need for ongoing monitoring of organ function, drug interactions, adherence, and bleeding/thrombosis [23,24,27,28,29,30]. The European Heart Rhythm Association guidelines recommend assessment of hemoglobin, liver function, and renal function at least annually for all patients. For patients with CrCl of 30 to 60 mL/min, those patients > 75 years, or fragile, they recommend more frequent evaluation of renal function every 6 months. For patients with CrCl of 15 to 30 mL/min, evaluation of renal function every 3 months should be considered [23]. Although there is no clear consensus regarding how and when to follow up, patients should be informed of a prespecified follow-up schedule.

## 6. Conclusions

Education for warfarin, a narrow therapeutic index medication, is routinely incorporated in the outpatient and inpatient setting. The need for INR monitoring often allows for greater access to health care providers and subsequently more education. The below list includes additional counseling points for patients on warfarin [43,44,45]:

- Regular INR tests are needed to ensure warfarin is working properly
- The goal INR range is often between 2 and 3; risk for clotting is greater when INRs are less than 2, risk for bleeding is higher when INRs are greater than 3; doses of warfarin are modified based on INR test results
- Each strength of warfarin has a unique color; with each refill make sure the tablets are the same color
- Foods with a lot of vitamin K like kale, collard greens, and spinach may interfere with warfarin; you do not need to avoid foods with vitamin K, but need to try to maintain consistent dietary habits on a weekly basis
- Alcohol increases the risk for bleeding and interferes with warfarin therapy; no more than 1–2 drinks per day, and avoid binge drinking

**Figure 1:**
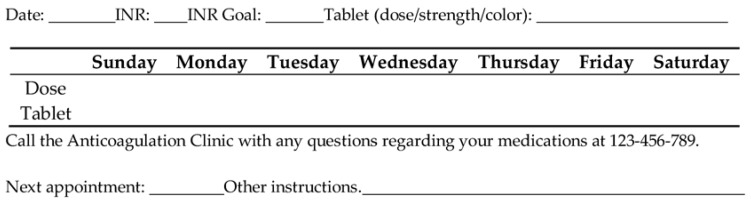
Sample Warfarin Dose Instruction Card.

Given the need to make adjustments to the dosing, it is recommended to provide written instructions for the patient, as shown in Figure 1 [43]. A wealth of evidence-based resources are available in assisting practitioners on how to effectively educate patients on warfarin [43,45].

Given the potential harm associated with anticoagulant use, patient education should be incorporated in a variety of pharmacy practice settings, including in ambulatory care clinics, hospital settings, and community pharmacies. The verbal face-to-face teaching sessions should be tailored to each patient, be accompanied with written resources and use the teach-back method.

## Figures and Tables

**Table 1:** Practical Considerations of Oral Anticoagulants [27,28,29,30].

Drug	Warfarin	Dabigatran	Rivaroxaban	Apixaban	Edoxaban
Dosing Frequency (For venousthromboembolism treatment or atrial fibrillation thromboprophylaxis)	Daily, adjusted based on INR	Twice daily	Daily or twice daily depending on indication	Twice daily	Daily
Missed Dose	Take if before midnight on the same day	Take as soon as possible (asap) on same day but at least 6 h before next scheduled dose	If missed a 15 mg tablet, take asap but can take two 15 mg tablets together. Patients on once daily regimen should take asap on same day.	Take asap on same day	Take asap on same day
Administration	With or without food	With a full glass of water; with or without food	With food	With or without food	With or without food
Weekly pill box	Can aid adherence	MUST store in original container and keep sealed.	Can aid adherence	Can aid adherence	Can aid adherence
Drug-Drug Interactions	Numerous; primarily via CYP2C9; minor pathways include CYP2C8, 2C18, 2C19, 1A2, and 3A4	Important drug:drug interactions: P-gp inducers and inhibitors (especially if renal function compromised)	Avoid dual P-gp and strong CYP 3A4 inducers or inhibitors	Avoid dual P-gp and strong CYP 3A4 inducers or inhibitors	Important drug:drug interactions: P-gp inducers and inhibitors
Can you crush?	Yes	No; Swallow whole; do not cut, open, or crush	Yes	Yes	Yes
